# Phase informed model for motion and susceptibility

**DOI:** 10.1002/hbm.22126

**Published:** 2012-06-27

**Authors:** Chloe Hutton, Jesper Andersson, Ralf Deichmann, Nikolaus Weiskopf

**Affiliations:** ^1^ Wellcome Trust Centre for Neuroimaging UCL Institute of Neurology University College London United Kingdom; ^2^ Oxford Centre for Functional MRI of the Brain FMRIB John Radcliffe Hospital Oxford University Headington Oxford United Kingdom; ^3^ Brain Imaging Centre Goethe University Frankfurt Germany

**Keywords:** EPI, distortion correction, dynamic field mapping, phase image, head motion, susceptibility artifact

## Abstract

Field inhomogeneities caused by variations in magnetic susceptibility throughout the head lead to geometric distortions, mainly in the phase‐encode direction of echo‐planar images (EPI). The magnitude and spatial characteristics of the distortions depend on the orientation of the head in the magnetic field and will therefore vary with head movement. A new method is presented, based on a phase informed model for motion and susceptibility (PIMMS), which estimates the change in geometric distortion associated with head motion. This method fits a model of the head motion parameters and scanner hardware characteristics to EPI phase time series. The resulting maps of the model fit parameters are used to correct for susceptibility artifacts in the magnitude images. Results are shown for EPI‐based fMRI time‐series acquired at 3T, demonstrating that compared with conventional rigid body realignment, PIMMS removes residual variance associated with motion‐related distortion effects. Furthermore, PIMMS can lead to a reduction in false negatives compared with the widely accepted approach which uses standard rigid body realignment and includes the head motion parameters in the statistical model. The PIMMS method can be used with any standard EPI sequence for which accurate phase information is available. Hum Brain Mapp 34:3086–3100, 2013. © 2012 The Authors. Human Brain Mapping Published byWiley Periodicals, Inc.

## INTRODUCTION

Echo‐planar imaging (EPI), the technique most commonly used for functional imaging studies, is particularly sensitive to inhomogeneities of the *B*_0_ field due to its low bandwidth in the phase‐encoding (PE) direction [Jezzard and Balaban, 1995]. Thus, field inhomogeneities caused by variations in magnetic susceptibility throughout the head lead to geometric distortions in the PE direction of EPI volumes which change with head position in the magnetic field [Jezzard and Clare, 1999]. Image distortion can lead to inaccurate registration with anatomical images and mislocalization of activation. Temporal changes in distortion caused by, for example, head movement, can lead to temporal signal fluctuations that remain after standard rigid body realignment procedures (e.g., [Friston et al., 1995]) have been applied. This residual variance can have a detrimental effect on the temporal SNR (tSNR) and hence fMRI studies which require maximal sensitivity to small BOLD signal changes. A widely accepted way of reducing motion related residual signal changes that remain after standard realignment is to include the estimated motion parameters in the statistical model as nuisance regressors [Friston et al., 1996]. Although this correlational approach often reduces noise and false positives, it can also lead to false negatives. As higher field strength scanners are used to acquire images of higher resolution (in the absence of acceleration factors such as parallel imaging), these temporal signal fluctuations will be accentuated due to an increase in local field differences coupled with longer EPI readout times and consequently lower PE bandwidths.

Several approaches have been proposed to correct for EPI distortions, e.g., [Chen and Wyrwicz, 1999; Jezzard and Balaban, 1995; Morgan et al., 2004; Reber et al., 1998; Zaitsev et al., 2004]. One of the most widely implemented methods to correct for distortions in EPI fMRI time‐series is based on the acquisition of a field map from which a map of distortions can be estimated [Jezzard and Balaban, 1995]. Since the magnitude and spatial characteristics of the distortions are dependent on the orientation of the head in the magnetic field [Jezzard and Clare, 1999], the movement‐related changes in distortion can not be corrected for using a single field map.

Previous studies have proposed methods to address the issue of the change in field (and hence distortion) due to head motion in EPI time series. These methods either require longer acquisition times to acquire additional echoes [Hutton et al., 2002; Weiskopf et al., 2005] or estimate the rate of change of the field with respect to subject movement directly from the magnitude signal change over the whole EPI time‐series [Andersson et al., 2001].

As proposed in [Jezzard and Clare, 1999], it is possible to estimate how much the field changes over the course of an EPI time series by calculating the difference in the phase between the first and successive images. This idea has been exploited for the purpose of performing dynamic distortion correction in single echo‐time EPI time series [Hahn et al., 2009; Hutton et al., 2005; Lamberton et al., 2007; Marques and Bowtell, 2005]. Common to the methods proposed in [Hahn et al., 2009; Lamberton et al., 2007; Marques and Bowtell, 2005] is the estimation of the dynamic field change from the measured phase at each time point which is then spatially modeled using polynomials.

In contrast to these methods, we have proposed an approach [Hutton et al., 2005] which fits a general linear model (GLM) [Friston, 2007] based on head motion parameters to the measured phase at each voxel in the single echo‐time EPI time series. By fitting a linear model to the phase time series at each voxel it is possible to estimate spatial maps of rate of change of phase with respect to head motion. These spatial parameter maps provide a correction for changes in distortion in the EPI magnitude data which are less sensitive to temporal noise in the phase compared with estimating the dynamic field change from each time point individually.

In this study we extend this Phase Informed Model for Motion and Susceptibility (PIMMS) to include knowledge about the linear change in phase caused by heating of the passive shims [Foerster et al., 2005] as well as the head motion parameters estimated from EPI magnitude data. Thus PIMMS can be used to estimate spatial maps of the rate of change of phase (and hence field change) with respect to head motion and scanner hardware. The maps of rate of change of distortion with respect to head movement are then used to correct for the change in distortion at each EPI volume with respect to the space of the first image in the time series.

We used three different proof of concept studies performed in four subjects (single subject and repetition design) to validate the PIMMS model and assess the impact of the correction. We acquired EPI time series during which the subjects were either instructed to make large systematic head movements (Experiment 1) or to remain still (Experiment 2) and during which a visual stimulus fMRI experiment was performed with and without small stimulus‐correlated head movements (Experiment 3). The fit of the PIMMS model to the phase data was assessed for all EPI time series. The impact of the PIMMS correction on the EPI magnitude time series was evaluated in terms of the reduction in movement‐related variance, the improvement in tSNR, and the reduction in false negative activations compared with processing using standard realignment procedures.

## METHODS

The theory behind the PIMMS approach is described in the next section, followed by details about the implementation and experimental application of PIMMS.

### PIMMS Theory

The PIMMS method is based on the following assumptions. We assume that phase changes occur due to heating of the passive shims [Foerster et al., 2005] as well as due to changes in head position and that the former can be modeled as a linear function of time. In addition, we assume that the observed phase changes are relatively small, vary linearly with head movements and are mainly caused by head rotations about the two axes that are nonparallel to the static magnetic field. Thus, we can describe local phase changes measured at each time point as a function of the respective motion parameters and approximate this relationship as a first order Taylor expansion. We also assume that all EPI volumes are aligned to the first image in the time‐series. This is approximated by including realignment into the PIMMS procedure.

### Taylor Expansion of the Change of Phase With Respect to Head Motion

The field experienced by the object and hence the measured local image phases depend on the subject's head position. In the following, we define the phase at a single voxel in an EPI volume measured at a time point *i* as ϕ*^i^*. If rigid body head motion is assumed, the current head position can be defined by a six‐dimensional vector **p** *^i^* (3 translation and three rotation parameters). If phase changes depend only on head motion, the difference between the phase measured at time points 1 and 2, and at corresponding head positions **p** ^1^ and **p** ^2^, can be approximated by a first order Taylor expansion:

(1) $$\varphi^{2}-\varphi^{1}\approx \sum_{n=1}^{6}\left(p_{n}^{2}-p_{n}^{1}\right){\frac{\partial \varphi }{\partial p_{n}}|}_{p^{1}}$$

It should be stressed here that the phase difference described in equation (1) is calculated on a voxel‐by‐voxel basis in the motion‐corrected frame of reference. We assume that the measured phase is mainly affected by rotations about the two axes that are nonparallel to the static magnetic field. For a normal supine or prone subject position, these are rotations about the right–left axis connecting the ears, and the anterior–posterior axis connecting the back of the head to the front. These axes will be referred to as the *x*‐ and *y*‐axis, respectively. If we define the rotation angle of these two types of head movement as θ*_x_* and θ*_y_*, and assume these are the only effects that give rise to changes in phase, then Eq. (1) can be simplified to contain just two terms. Furthermore, it can be generalized to describe the phase change between any point *i* in the time series and the first one as long as the movements are small and the linear approximation is valid:

(2) $$\varphi^{i}-\varphi^{1}\approx \left(\theta_{x}^{i}-\theta_{x}^{1}\right){\frac{\partial \varphi }{\partial \theta_{x}}|}_{\theta_{{}_{x}}^{1},\theta_{{}_{y}}^{1}}+\left(\theta_{y}^{i}-\theta_{y}^{1}\right){\frac{\partial \varphi }{\partial \theta_{y}}|}_{\theta_{{}_{x}}^{1},\theta_{{}_{y}}^{1}}$$

### Estimating Rate of Change of Phase With Respect to Motion Using the GLM

The terms of the first order Taylor expansion in Eq. (2) can be reformulated in a GLM framework [Friston, 2007] as:

(3) Y=Xβ+ɛ

In Eq. (3), *Y* is the column vector of observations, in this case, the measured change of phase with respect to the first image in the time series, *Y* = Δϕ = [(ϕ^2^ − ϕ^1^) ··· (ϕ^*N*^ − ϕ^1^)]^T^ and ε is the column vector of error terms. X is the design matrix which contains one row per observation and one column or explanatory variable per model parameter, where the explanatory variables are vectors describing the changes in rotation of the head about the *x* and *y* axes, Δθ*_x_* = [(θ_*x*_^2^ − θ_*x*_^1^),…(θ_*x*_^*N*^ − θ_*x*_^1^)]^T^ and Δθ*_y_* = [(θ_*y*_^2^ − θ_*y*_^1^),…(θ_*y*_^*N*^ − θ_*y*_^1^)]^T^ respectively. β is the column vector of *L* model parameters, β = [β*_1_,…,β_L_*]^T^ to be estimated. In this case *L* = 2 and β corresponds to the rate of change of phase with respect to motion, such that β*_1_* corresponds to ∂ϕ/∂θ_x_ and β*_2_* corresponds to ∂ϕ/∂θ_y_.

### Extension of the Model to Include Shim Heating Effects

We know that a linear phase change or drift is often observed during the acquisition of EPI time series. This leads to an apparent translation of the imaged object along the phase‐encode direction in the magnitude images. This linear change in phase is attributed to the heating of the ferromagnetic passive shims caused by mechanical vibrations between parts of the MRI scanner due to the rapidly switched gradients during the EPI acquisition [Foerster et al., 2005]. Although this effect does not arise from true head motion, the component of the phase which is spatially invariant causes a rigid‐body translation in the magnitude image time series and can therefore be corrected using rigid body realignment. However, the phase change persists in the phase data after motion correction and must therefore be taken into account in the PIMMS model so as not to interfere with the estimation of the other parameters. To do this, a linear term Δ*t* = [(*t* ^2^ − *t* ^1^),…,(*t* ^*N*^ − *t* ^1^)] representing the linear phase drift over time *t* caused by scanner shim heating is included in the model as an additional variable. A constant term K comprising of a column of ones is included to model the mean of the measured change in phase over the time series.

The design matrix (from equation (3)), extended to include shim heating effects, is therefore formed by X = [Δθ*_x_* Δθ*_y_* Δ*t* Κ]. To ensure that X contains a set of orthogonal basis functions a Gram–Schmidt process is used to orthogonalize the columns of X from right to left resulting in the transformed design matrix X′ = [θ*_x_* θ*_y_* τ Κ]. Solving equation (3) but with the transformed design matrix results in estimates for each of the four model parameters β, i.e., β*_1_* and β*_2_* correspond to rate of change of phase with respect to head rotation about the x and y axes (∂ϕ/∂θ*_x_* and ∂ϕ/∂θ*_y_*), β*_3_* corresponds to rate of change of phase with respect to time (∂ϕ/∂τ) and β*_4_* to the mean change in phase over the whole time series (C).

*Using the rate of change of phase to calculate the PIMMS correction*

Using the estimated rate of change of phase maps (i.e., β = [β*_1_,…, β_L_*], *L* = 4), the modeled change in phase relative to the first image, Δϕ_mod el_^*i*^ can be constructed for each image *i* in the time series by multiplying the estimated β by each row of the orthogonalized design matrix X′:

(4) $$\Delta \Phi_{\text{model}}^{i}=\Theta_{x}^{i}\beta_{1}+\Theta_{y}^{i}\beta_{2}+\tau^{i}\beta_{3}+K\beta_{4}$$

Equation (4) provides a model for the local phase change at each time point. The third term in Eq. (4), which is primarily concerned with modeling shim heating effects, describes the linear change in phase with respect to time. The spatially invariant component of this term causes an apparent translation of the head in the EPI magnitude images which is corrected using rigid body realignment. To avoid correcting for this effect twice, (i.e., once by the rigid body realignment and once by the PIMMS correction) the PIMMS correction is calculated using the following:

(5) $$\Delta \Phi_{\text{correction}}^{i}=\Delta \Phi_{\text{model}}^{i}-\Delta t\bar{\beta_{3}}$$

where $\Delta t\bar{\beta_{3}}$ represents the spatially invariant component of the linear phase change. Note that the relationship between the orthogonalized linear regressor τ and Δ*t* is given by τ = $\Delta t-\bar{\Delta t}$. The PIMMS correction Δϕ_correction_^*i*^ therefore corrects only for spatially varying changes in phase resulting from head motion or spatially inhomogeneous off‐resonance effects due to shim heating and does not correct for rigid body translations in the EPI magnitude images.

### Performing the Dynamic Distortion Correction

From Eq. (5), the resulting map of local phase changes associated with each time point *i* can be scaled from radians to a change in *B*_0_ field, ΔΒ*_0_^i^* in Hz by dividing by the echo time, TE, of the EPI volume, and a value of 2π since the phases are given in radians (i.e., ΔΒ*_0_^i^*= Δ ϕ_correction_^*i*^/(2π TE)). ΔΒ*_0_^i^* must be divided by the bandwidth per pixel in the phase‐encode direction, BW_PE,_ to give the relative distortion in voxels, resulting in a voxel displacement map (vdm):

(6) $${\text{vdm}}^{i}=\Delta B_{0}^{i}/BW_{\text{PE}}$$

Each vdm*^i^* describes the voxel displacement in the phase encode direction caused by the change in field at each voxel in the distorted image *i* with respect to the space of the first image. To calculate the field required to correct for the relative distortion between each image and the first in the time series, the vdm*^i^* must be inverted. For this inversion procedure, the forward mapping gives us *x′* = *f*(*x*) for each value x on a regular grid, where *x* is a 1‐dimensional column in the phase encode direction. The inverse mapping yields *x* = *f* ^−1^(*x*′) for each value *x*′ on a regular grid. It is calculated by, for each value *x*′, finding the first *x*_*i*_ so that *f*(*x*_*i*_) > *x*′ and then finding *x* by linear interpolation between *f*(*x*_*i*−1_) and *f*(*x*_*i*_). This is a valid procedure as long as *f*(*x*) is monotonously increasing, which is the case in general. When it is not, i.e., when the off‐resonance field changes very rapidly over space (compared with the effective encoding gradient strength), there will be almost total signal loss in the gradient echo EPI images, so the displacement of the nonexistent signal will not matter. Finally, at each time point *i*, the original distorted image can be distortion corrected to the space of the first image by resampling distorted image voxels at new locations along the phase encode direction according to the values in the vdm*^i^* [Hutton et al., 2002; Jezzard and Balaban, 1995].

### PIMMS Implementation

**Figure 1:**
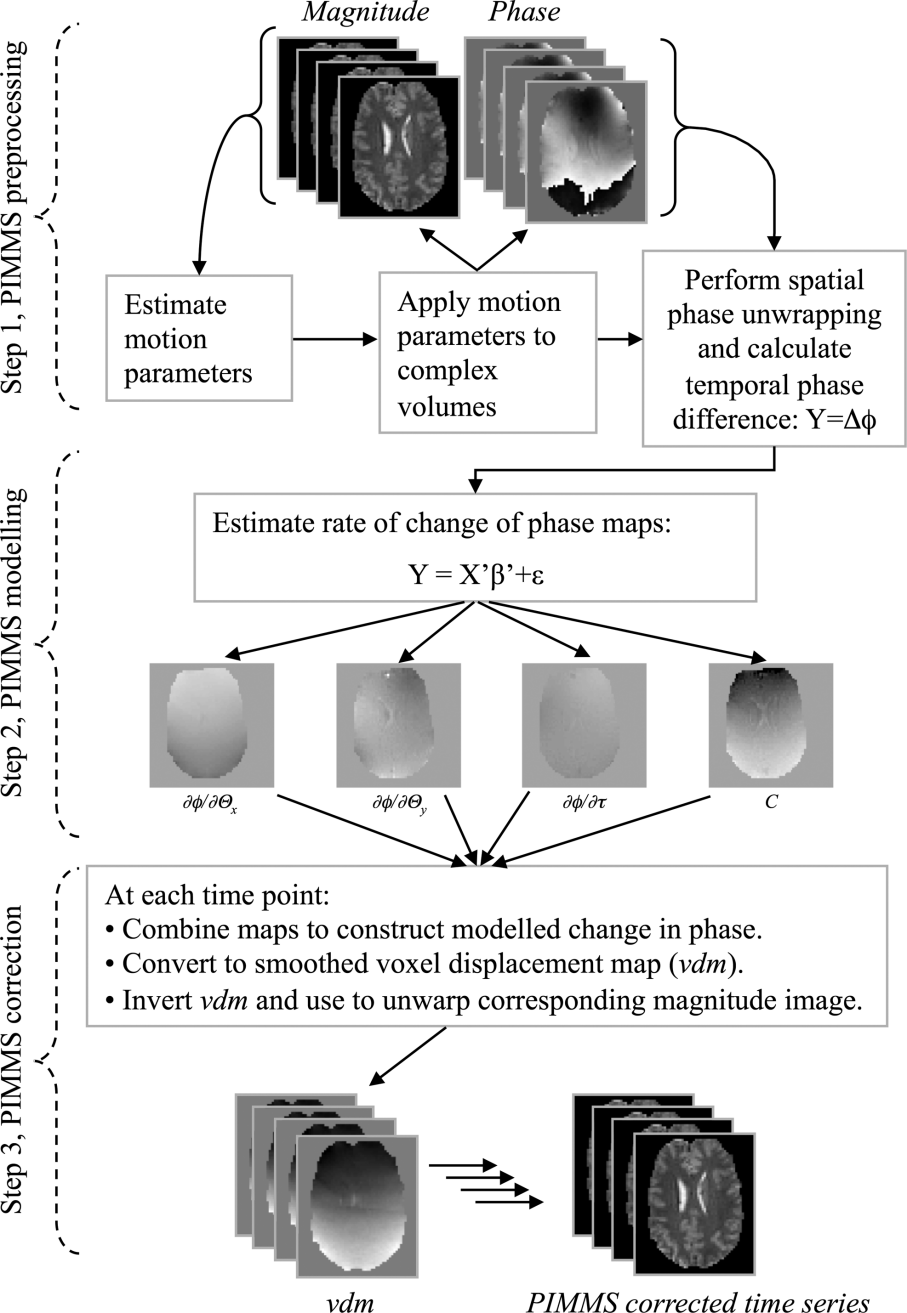
Diagram illustrating the different steps for the PIMMS implementation. See sub‐section “PIMMS Implementation” in the Methods section for a description of the steps.

The PIMMS implementation comprised three steps which were preprocessing, modeling, and correction. These are outlined in Figure 1 and described in the following sections.

#### Step 1, PIMMS preprocessing

Magnitude images from complex EPI time series (consisting of both phase and magnitude data) were realigned to the first image in the time series using the rigid body model implemented in SPM8 (http://www.fil.ion.ucl.ac.uk/spm/, [Friston et al., 1995]) . Since the measured phase can take only values in a 2π range, phase discontinuities occur in the phase images. The original phase values can be recovered by adding or subtracting multiples of 2π where the phase jumps occur and this can be done robustly within a three‐dimensional phase image using a “phase unwrapping” algorithm [Jenkinson, 2003]. Furthermore, phase discontinuities may also occur across time points throughout an EPI time series. In this implementation, phase discontinuities were first removed from each phase volume in the time series by applying three‐dimensional phase unwrapping. Each unwrapped phase volume was then resampled into the space of the first image using the estimated motion parameters. A mask was generated from the first wrapped phase image in the time series and used to exclude the background noise from all unwrapped phase difference images in the time series. To generate the mask, the angular variance of the wrapped phase was thresholded at π^2^/6 radians. This threshold value was determined by estimating the variance of noise from the joint histogram of real and imaginary parts of the image. In general this threshold will depend on factors such as field strength and voxel size which affect image SNR. Finally, the voxelwise phase difference (Δϕ) between each time point and the first one was then calculated (i.e., Y = [(ϕ*^2^ − ϕ^1^*) …(ϕ*^N^ − ϕ^1^*)]).

#### Step 2, PIMMS modeling

The PIMMS model was constructed using the GLM framework and Eq. (4) as described in the PIMMS theory section. Solving the GLM resulted in four spatial maps of model fit parameters describing the rate of change of phase associated with head rotations about the *x* and *y* axes (∂ϕ/∂θ*_x_* and ∂ϕ/∂θ*_y_*), the rate of change of phase with time (∂ϕ/∂τ) and the mean phase change over the time series (C).

#### Step 3, PIMMS correction

The PIMMS correction for change in distortion at each image with respect to the first image in the time series was constructed using the estimated rate of change of phase maps and Eq. (5). The signal‐to‐noise ratio of each PIMMS correction map was increased a little by smoothing with a Gaussian kernel of FWHM = 3 mm. The resulting map was scaled using Eq. (6) to yield a voxel displacement map (vdm). Each vdm was inverted and used to resample the corresponding EPI magnitude image resulting in an image which was corrected for distortions relative to the space of the first image in the time series.

The PIMMS routines were implemented in Matlab (The MathWorks, Natick, MA) version 7.8 using routines from SPM8 [Friston, 2007] and the SPM8 FieldMap toolbox [Hutton et al., 2004].

### Experimental Application of PIMMS

Four healthy volunteers were scanned with written informed consent on a 3T head scanner (Magnetom Allegra, Siemens Healthcare, Erlangen, Germany) with a head transmit‐receive RF coil. Approval for the study was obtained from the local ethics committee. Three different experiments were performed to test the experimental application of PIMMS. Two of the four subjects performed all experiments, one subject performed only one experiment and one subject performed two experiments. This resulted in data from three subjects for each experiment.

### Experiment 1—EPI Time Series With Systematic Head Movement

In Experiment 1, single shot, gradient echo EPI time series were acquired during which the subjects were visually cued to tilt their head forwards (about ± 2–3° around the *x*‐axis) and then cued to tilt it back to the original position. The total cycle length for the two head positions was 56 s which was repeated nine times. EPI data were acquired with the following sequence parameters: matrix = 64 × 64, resolution = 3 × 3 mm^2^, 32 slices, thickness = 2 mm + 1 mm gap, TE = 30 ms, bandwidth in the phase‐encoding direction BW_PE_ = 31.25 Hz/pixel. Each EPI volume was acquired with a TR of 2.08 s plus a pause of 5.92 s, resulting in a total interval of 8 s between the start of subsequent volume acquisitions. The pause was inserted between each volume acquisition so that the subjects could move their heads as instructed during the periods when no data were being acquired, avoiding intrascan motion. Each EPI time series comprised 63 complex volumes.

### Experiment 2—EPI Time Series Without Stimulus or Head Movement

In Experiment 2, subjects were instructed to rest with their eyes open and without moving for ∼8 min. For this experiment, EPI data were collected with the same parameters as in the first experiment but using a TR of 2.08 s (i.e., with no pause between volumes) and with a bandwidth in the phase‐encoding direction = 47.35 Hz/pixel. In contrast to Experiment 1, acquisition parameters were chosen which are more typical for fMRI studies. Each EPI time series comprised 144 complex volumes.

### Experiment 3—Visual Stimulus fMRI With and Without Head Movement

In Experiment 3, EPI time series were acquired during which the subjects were visually presented with 25 s of an alternating checkerboard (frequency of 8 Hz) versus 25 s of a blank screen. The cycle length of 50 s was repeated six times. The EPI run was performed twice, once with head movement and once without. In the first run, ∼6 s after the start of the checkerboard presentation, the subjects were cued to tilt their head forwards by a small amount (<1.0° around the *x*‐axis) and then tilt it back to the original position after presentation of the blank screen. The cue was a subtle change in the background of the visual stimulation and was present in runs with and without head movement. The timing of the cue was chosen to maximize the chance of head movement being correlated with the hemodynamic response to the stimulus. In the runs without head movement, the subjects were instructed to remain still. EPI data were collected with the same parameters as in Experiment 2.

### Data Processing

For all experiments, complex data volumes were acquired and used to reconstruct phase and magnitude images using a trajectory‐based reconstruction designed to minimize ghosting [Josephs et al., 2000]. Each EPI time series was preprocessed using two different methods followed by smoothing using a Gaussian kernel with FWHM = 6 mm. For Method A, the standard rigid body realignment and reslicing procedure as implemented in SPM8 [Friston et al., 1995] was applied to the magnitude images only (i.e., no correction for dynamic distortion effects). For Method B, the PIMMS modeling and correction procedure was applied to phase and magnitude images. The processing incorporated realignment, estimation of PIMMS parameter maps from the phase data and dynamic distortion correction as described previously and outlined in Figure 1. As a final step, the PIMMS corrected data were realigned and resliced (as in Method A) to account for any inaccuracies in the initial realignment step caused by the changes in distortion throughout the time series.

### Validation of PIMMS Model

The PIMMS model was validated by assessing the fit of the model to the phase data acquired in each EPI time series for all experiments. The model fit was determined by calculating the F‐statistic at each voxel in the image volume. The location and percentage of voxels in the brain where a significant amount of variance was explained by the model was calculated. A probability value of *p* < 0.001 was considered to be significant.

To check the assumption that the measured phase was mainly affected by rotations about the two axes that are nonparallel to the static magnetic field, a second PIMMS model was constructed that included all six of the estimated head motion parameters (i.e., three translations and three rotations). This model was fitted to each EPI time series and assessed by calculating the F‐statistic at each voxel in the image volume. The results were compared with the first model in terms of percentage of voxels in the brain showing a significant fit of the model, and change in residual error. Note that no further processing or analyses were performed using this second PIMMS model.

The data acquired in Experiment 1 was used to characterize the spatial distribution and magnitude of phase changes explained by the original PIMMS model. The average phase change and model fit over all voxels in the brain were calculated at each time point to demonstrate the fit of the model over time. Spatial maps were calculated where the value at each voxel was the percentage of the total standard deviation of phase changes explained by the PIMMS model in Eq. (4). Finally, for each subject, a spatial map of the mean of the absolute modeled voxel displacement over the time series (i.e., |vdm|) was calculated.

### Evaluation of PIMMS Correction

The data acquired for Experiment 1 and Experiment 2 (i.e., experiments without a specific stimulus but with and without head movement respectively) were used to evaluate the impact of the PIMMS correction on tSNR. For this, tSNR maps (tSNR_A_ and tSNR_B_) were calculated by dividing the mean of each voxel time series by the standard deviation. The tSNR maps for the PIMMS preprocessing (Method B) were compared with those for the realignment (Method A) using (tSNR_B_/tSNR_A_)‐1.

The data acquired from Experiment 1 were used to compare the residual magnitude signal changes associated with head movement between data processed using Methods A and B. For this comparison, the processed data were entered as two sessions into a single GLM in SPM8 which comprised of a regressor describing the estimated head rotations about the *x*‐axis (i.e., the main axis of instructed head motion). Voxel‐wise *t*‐tests were used to detect voxels where the magnitude of the parameters describing residual motion effects were significantly greater for either data processed using Method A or Method B. Voxels with a probability value of *p* < 0.05 corrected for family wise errors over the brain using random field theory [Worsley et al., 1996] were considered to show significant differences.

The data acquired from Experiment 3 were used to compare the fMRI BOLD effects detected in the EPI magnitude images processed using Methods A and B for each subject. For this comparison, for each run, the processed data were entered as two sessions into a single GLM which comprised of a regressor describing the visual stimulus timing. Voxel‐wise *t*‐tests were used to detect voxels where the magnitude of the parameters explaining the visual response were significantly greater for either data processed using Method A or Method B. Voxels with a probability value of *p* < 0.05 corrected for family wise errors were considered to show significant differences.

Experiment 3 comprised of two fMRI runs, one with and one without stimulus‐correlated head movement. Our assumption was that the detection of BOLD (blood oxygen level dependent) responses to the visual stimulus would be optimal (i.e., minimal false positives or false negatives) in the run without head movement, and for this run the fMRI results would not be different between data processed using Methods A and B. We also assumed that including motion parameters in the GLM may lead to false negatives when visual task and head motion were correlated. Therefore, by comparing the results between the different processing methods and the runs with and without head motion, it was possible to estimate whether the PIMMS correction lead to a reduction in false negatives compared with using standard rigid body realignment including motion parameters in the GLM (Method A+MP).

For this comparison, data processed using Method B were entered into a GLM comprising a single visual stimulus timing regressor and data processed using Method A were entered into a GLM comprising the six motion parameters in addition to the visual stimulus timing regressor. Voxel‐wise *t*‐tests were used to detect voxels where the BOLD response was greater for the alternating checkerboard compared to the blank screen. For each analysis and experimental run, the number of significantly activated voxels (i.e., with *p* < 0.05 corrected for family‐wise errors over the brain) was counted within a visual cortex region of interest (vcROI). The vcROI was defined anatomically according to the brain atlas provided with the AAL toolbox [Tzourio‐Mazoyer et al., 2002] and nonlinearly matched to each subject space using SPM8 (as described in [Hutton et al., 2011]).

## RESULTS

The PIMMS processing steps included realignment, calculation of temporal phase difference, phase unwrapping, model fitting, and use of model fit parameter maps to resample and distortion correct the original magnitude images (PIMMS correction). Without any special optimization for speed, the PIMMS processing took approximately five times longer than standard realignment, with 70% of this time required for phase unwrapping which could be made faster.

### Validity of PIMMS Model

For all subjects, more than 50% of total phase variance was explained by the PIMMS model in more than 85% of voxels in the brain for Experiment 1 and more than 93% for Experiments 2 and 3. Correspondingly, the F‐statistic was significant in more than 84% of voxels for Experiment 1 and more than 94% of voxels for Experiments 2 and 3. For the second PIMMS model comprising all six of the estimated motion parameters, significant variance was explained in the same percentage of voxels and the mean and variation of residual errors were of the same order as the first PIMMS model for all EPI time series. These results suggested that for the data presented here, the fit of the PIMMS model was not improved by including the additional motion parameters.

**Figure 2:**
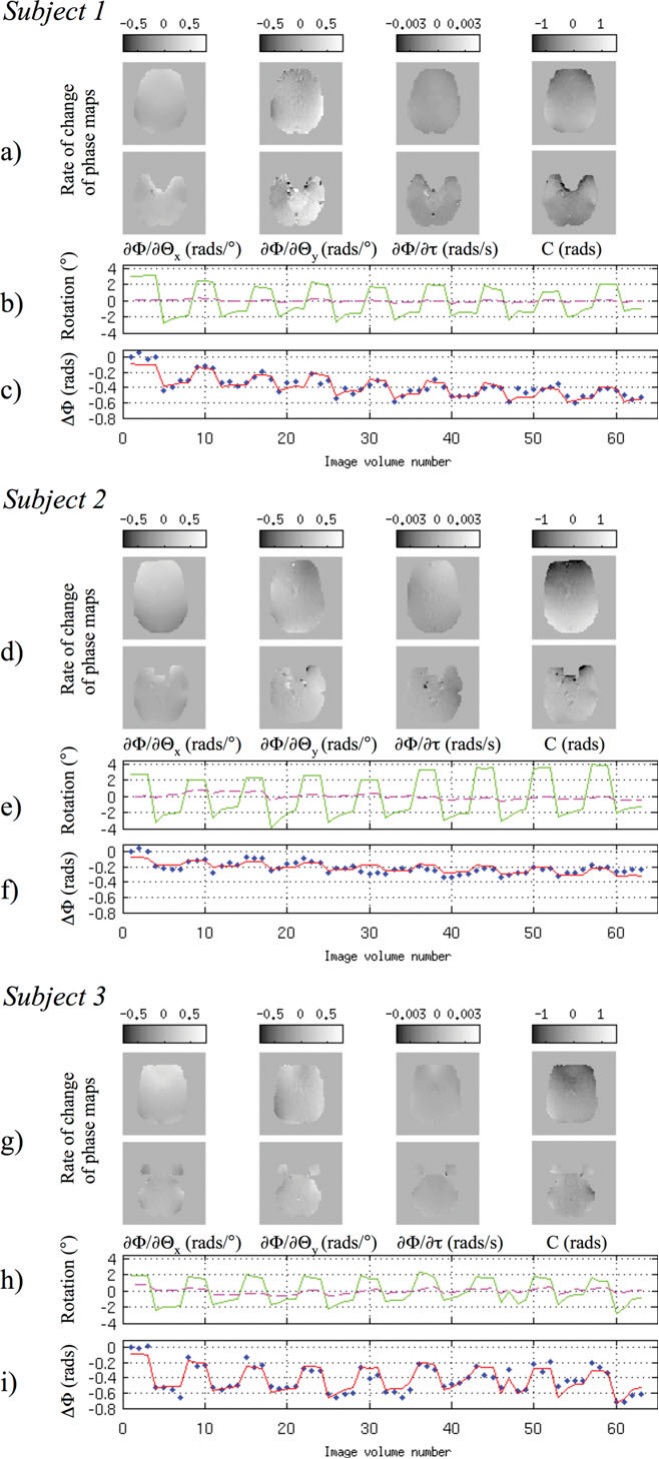
Fit of the PIMMS model to the change of phase data acquired in Experiment 1 for three subjects. Top rows (**a, d, g**), from left: Example slices through brain of rate of change of phase per degree of rotation about the *x* and *y* axes, ∂ϕ/∂θ_x_ and ∂ϕ/∂θ_y_ in radians per degree, rate of change of phase per unit time, ∂ϕ/∂τ in radians per second and the mean phase change C in radians. Middle rows (**b, e, h**): Head rotation estimated from realignment about the *x*‐axis (green solid line) and *y*‐axis (pink dashed line) in degrees versus image volume number. Bottom rows (**c, f, i**): Average phase change (blue dotted line) and the average model fit (red solid line) over all voxels in the brain (in radians) versus image volume number. [Color figure can be viewed in the online issue, which is available at http://wileyonlinelibrary.com.]

The fit of the PIMMS model to the phase data acquired in Experiment 1 is illustrated in Figure 2 for each subject. The top rows (a, d, g) show two slices (one inferior and one more superior) through each of the four rate of change of phase maps. From the left these are the rate of change of phase per degree of rotation about the *x* and *y* axes, ∂ϕ/∂θ*_x_* and ∂ϕ/∂θ*_y_* in radians per degree, rate of change of phase per unit time, ∂ϕ/∂τ in radians per second and the constant term C representing the mean phase change over the time series with respect to the first image. The middle rows (b, e, h) show a plot of the motion parameters estimated from the realignment for head rotation about the *x*‐axis (green solid line) and the *y*‐axis (pink dashed line) in degrees versus image volume number. The bottom rows (c, f, i) show the average phase change (blue dotted line) and the average model fit (red solid line) over all voxels in the brain in radians versus volume number. The impact of head rotation around the *x*‐axis is visible in the ∂ϕ/∂θ*_x_* maps which show an increased field at the front of the head and a decreased field at the back of the head. This means that head rotation about the *x*‐axis will lead to stretching of the front of the image in the anterior direction and of the back of the head in the posterior direction. For the ∂ϕ/∂θ*_y_* maps, there is left right asymmetry indicating the impact on the field of a head rotation about the *y*‐axis. The ∂ϕ/∂θ_x_ and the ∂ϕ/∂θ_y_ maps are shown at the same scale of ±0.5 radians per degree of rotation but it is apparent that the relative effect of rotation about the *y*‐axis is greater and the ∂ϕ/∂θ_y_ map contains more spatial structure. This is likely to be a result of there being very little head motion about the *y*‐axis resulting in a noisier estimate for this model parameter.

The motion parameters in the middle rows (b, e, h) demonstrate that estimated rotations were between ±2 and 3° around the *x*‐axis and up to ±0.5° about the *y*‐axis. Multiplying these values by the rate of change of phase estimates and scaling using Eq. (6) results in voxel shifts of up to 0.5 voxels (i.e., 1.5 mm) associated with the corresponding head rotations. The rate of change of phase per unit time, ∂ϕ/∂τ is scaled between ±0.003 radians which corresponds to a voxel shift of around 0.5 voxels (1.5 mm) over the experimental run. One would expect this effect to be spatially homogeneous but some structure can be observed in the rate of change of phase with time map. The mean phase change, C corresponds to a static distortion over the time series of between ±0.2 voxels. From the bottom plots (c, f, and i) it can be observed that on average over the brain, the estimated change of phase effects range from 0.2 to 0.6 radians which correspond to voxel shifts of 0.1–0.3 mm. The correspondence between the blue dotted and red solid lines illustrate the fit of the PIMMS model to the change of phase averaged over the whole brain.

**Figure 3:**
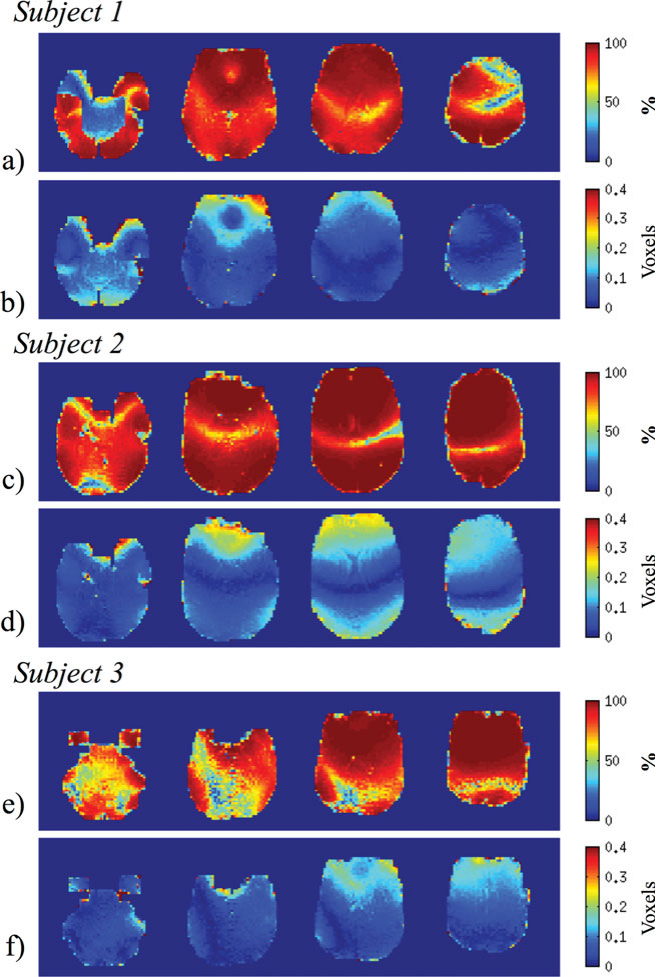
Magnitude and spatial characteristics of effects modeled by PIMMS for example slices through the brains of three subjects scanned in Experiment 1. Top rows (**a, c, e**): Percentage of the total phase change explained by the PIMMS model. Bottom rows (**b, d, f**): Mean of absolute voxel displacements estimated using PIMMS model. [Color figure can be viewed in the online issue, which is available at http://wileyonlinelibrary.com.]

Figure 3 shows the percentage of the total phase change modeled by PIMMS and the estimated mean voxel displacements over the brain in example slices for the three subjects scanned in Experiment 1. The slices in the top rows in Figure 3a,c,e show that for all subjects the PIMMS model explained a smaller percentage of variance along a central left–right oriented band in the brain. A larger percentage of variance was explained at the edges of the brain and close to borders between air and bone such as the nasal cavities. These results are also consistent with those shown in the bottom rows in Figure 3b,d,f which indicate the mean amount of local voxel displacement for the same slices in the brain. Larger voxel displacements occurred around the edges of the brain, in frontal regions and near the sinuses (up to 0.4 voxels, i.e., 1.2 mm). Smaller voxel displacements occurred in the central regions of the brain. These results demonstrate that head motion lead to larger phase changes in regions where the field was less homogeneous.

### Impact of PIMMS Correction

**Figure 4:**
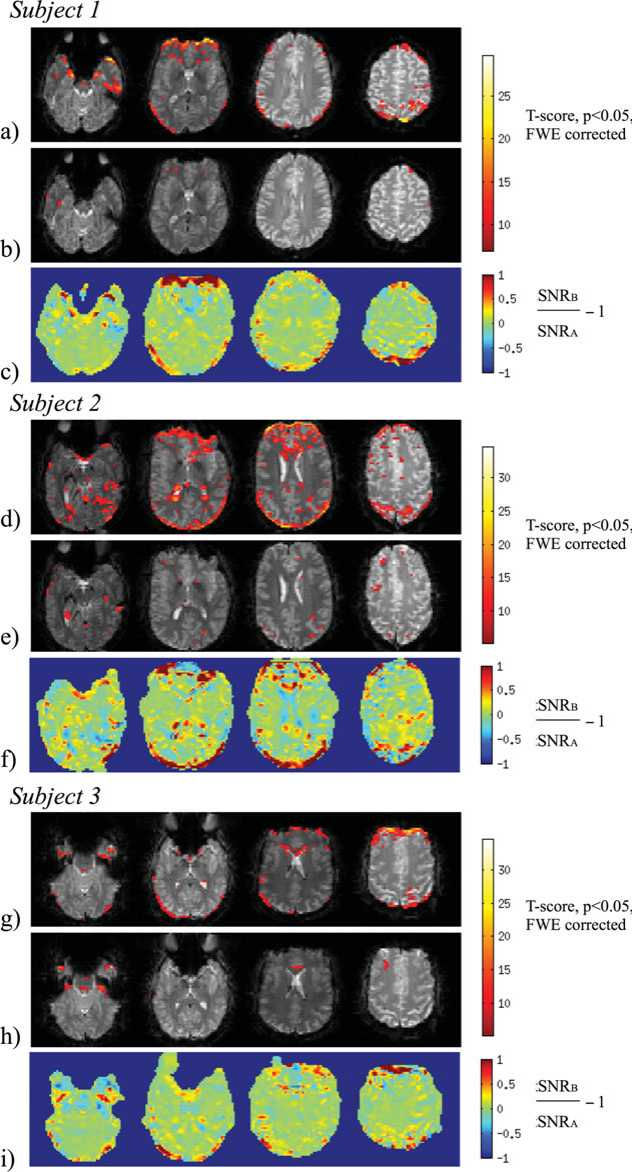
Impact of PIMMS correction shown for example slices through the brains of three subjects scanned in Experiment 1. Top two rows (**a, d, g**) and (**b, e, h**): Map of *t*‐scores overlaid on an EPI slice indicating where the magnitude of the model fit parameters for residual effects of head motion around the *x*‐axis are greater for the realigned data compared with PIMMS correction (top rows, **a, d, g**) and vice versa for the middle rows (**b, e, h**). The *t*‐scores are shown thresholded at a *p*‐value of 0.05 (FWE corrected). Bottom rows (**c, f, i**): Comparison between the tSNR of the data processed using PIMMS (Method B) and standard realignment (Method A), using tSNR_B_/tSNR_A_‐1 (masked with thresholded brain image). [Color figure can be viewed in the online issue, which is available at http://wileyonlinelibrary.com.]

**Figure 5:**
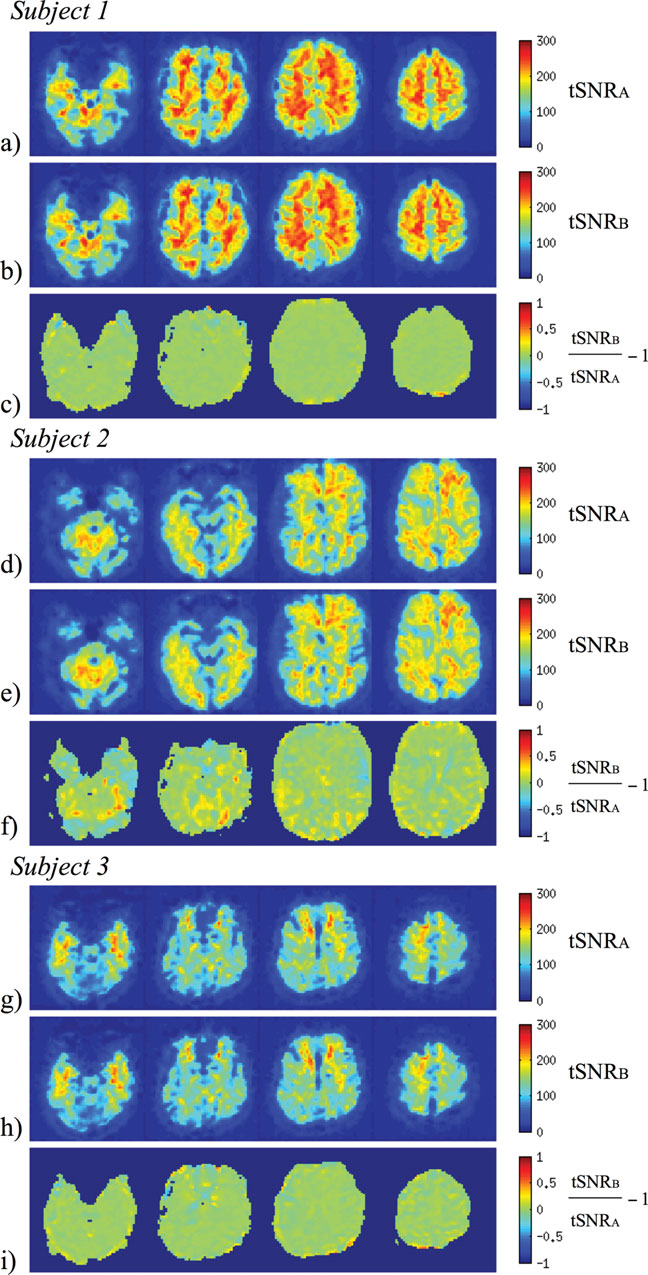
Impact of PIMMS correction shown for example slices through the brains of three subjects scanned in Experiment 2. Top two rows (**a, d, g**) and (**b, e, h**): tSNR maps for data processed using Method A (tSNR_A_) and Method B (tSNR_B_). Bottom rows (**c, f, i**): Comparison between the tSNR of the data processed using PIMMS (Method B) and standard realignment (Method A), using tSNR_B_/tSNR_A_‐1 (masked with thresholded brain image). [Color figure can be viewed in the online issue, which is available at http://wileyonlinelibrary.com.]

The impact of the PIMMS correction on tSNR is demonstrated for each subject in the bottom rows of Figure 4c,f,i for Experiment 1 and Figure 5 for Experiment 2. For Experiment 1, the comparison between tSNR maps for the data processed using PIMMS (tSNR_B_) and standard realignment (tSNR_A_) showed that tSNR_B_ improvement was more than 100% in regions maximally affected by motion‐related distortions, i.e., at the edges of the brain and close to tissue borders. In other parts of the brain, differences in tSNR of around ±25% were observed which mostly followed borders between gray and white matter and ventricles. For Experiment 2, minimal motion‐related distortion effects were expected and in contrast to Experiment 1, differences between tSNR_A_ and tSNR_B_ were observed around the edges of the brain and tissue boundaries of around ±25% in Subjects 1 and 3 and up to 50% in Subject 2. For both experiments, regions showing differences in the tSNR ratio (tSNR_B_/tSNR_A_‐1) of the order of about ±25% can be explained by relative displacements between different tissues in the magnitude images resulting from the two processing methods, (note that PIMMS processed data have been nonlinearly corrected for distortions relative to the space of the first image). It is clear from the top two rows for each subject in Figure 5(a,d,g and b,e,h) that the tSNR of white matter, gray matter, and CSF are very different (around 200, 150, and 100, respectively). Therefore even a subvoxel mismatch between tissue types in the maps of tSNR_A_ and tSNR_B_ could give rise to differences of around ±25% or more.

The impact of the PIMMS correction on the residual magnitude signal changes associated with head movement is demonstrated in Figure 4, in the top two rows for each subject (a, d, g and b, e, h). Results are shown in example slices for the three subjects scanned in Experiment 1 and correspond to those shown in Figure 3. The results indicate where the magnitude of residual motion effects is significantly greater for the realigned data compared with PIMMS corrected data (top row) and vice versa (middle row). The top row identifies regions where the PIMMS correction significantly reduced residual motion‐related variance compared with the standard realignment. In all subjects, the strongest differences are clearly around the front and back edges of the brain corresponding to regions where maximal image stretching and compression occurs as a result of the head rotation about the x‐axis. Significant effects can also be seen around the ventricles and along tissue boundaries. The results in the middle rows (b, e, h) indicate that the magnitude of residual motion effects are significantly greater for the PIMMS corrected data in small regions only which are mostly isolated voxels compared with the realigned data.

**Figure 6:**
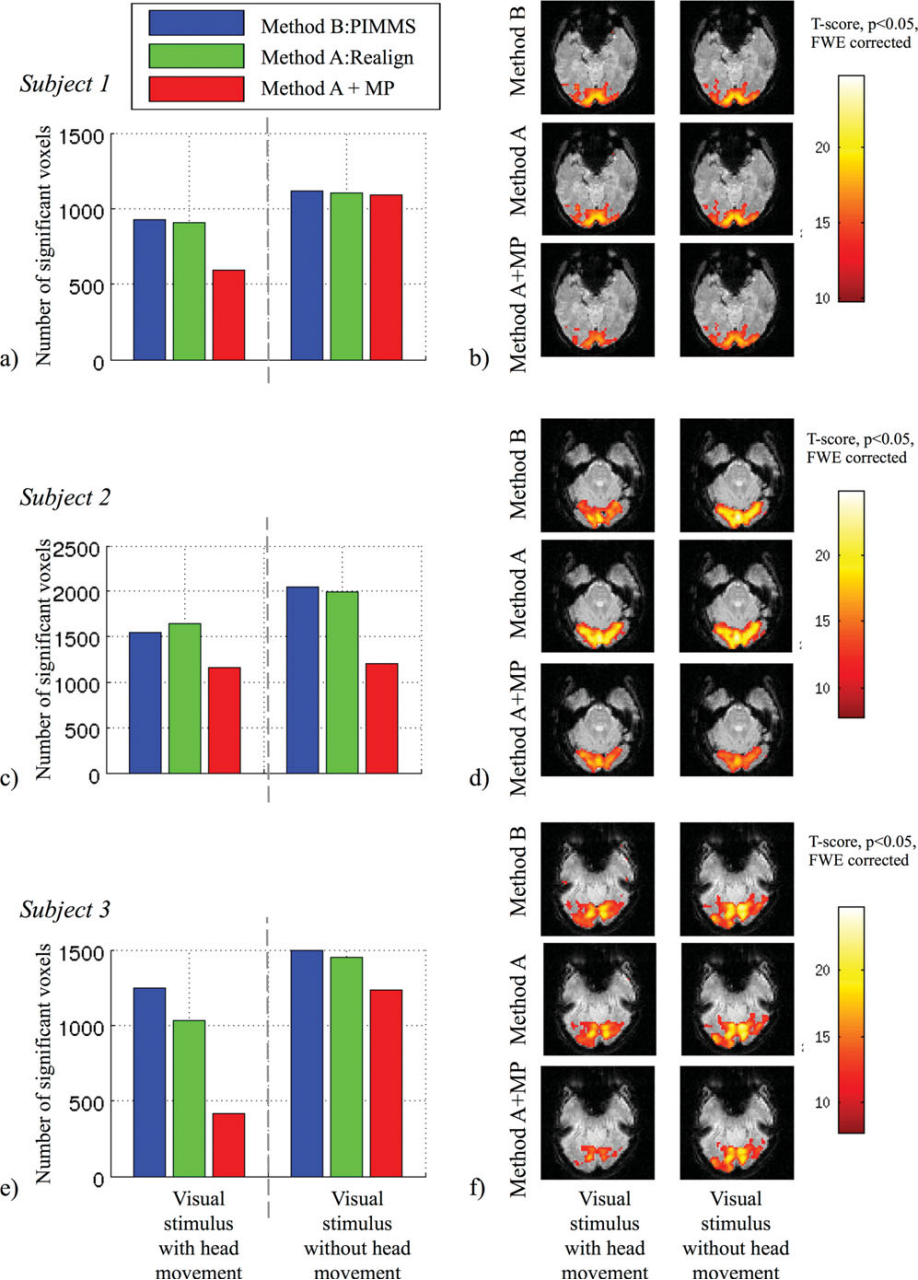
Impact of PIMMS correction on fMRI data acquired in three subjects in Experiment 3. Graphs on the left show the numbers of significantly activated voxels (*p* < 0.05 corrected for family wise errors) in the visual cortex ROI for fMRI runs with and without stimulus‐correlated head movement and for different processing methods (Method B: PIMMS, Method A: Realign and Method A+MP, i.e., with motion parameters included in the GLM). Images on the right show statistical maps of significant *t*‐scores representing visual cortex response to visual stimulus for different fMRI runs and different processing methods. [Color figure can be viewed in the online issue, which is available at http://wileyonlinelibrary.com.]

For the data acquired in Experiment 3, there were no significant differences between the magnitude of the parameters explaining the visual response for data processed using Method A and Method B, for both fMRI runs, with and without head movement. The graphs on the left of Figure 6 illustrate the similarity between the numbers of significantly activated voxels detected for data processed using Method A and Method B.

The fMRI data were also used to determine whether the PIMMS correction lead to a reduction in false negatives compared with using standard rigid body realignment, which included motion parameters in the GLM (Method A+MP). In Figure 6, a bar chart for each subject shows that the number of significantly activated voxels within a visual cortex ROI was reduced for all processing methods for the run with head movement (i.e., comparing bars on the left with bars on the right), as expected. The results also showed that the biggest reduction in significantly activated voxels was for Method A+MP (red bars) and this reduction was greater for the run with head movement. If we assume that the run without head movement should yield optimal fMRI results with minimal false positives and false negatives, we can attribute a reduction in activated voxels between runs with and without head motion to an increase in false negatives. Using the same arguments and the assumption that including motion parameters in the GLM may lead to false negatives, a reduction in activated voxels detected for data processed using Method A+MP can be attributed to an increase in false negatives compared with using the PIMMS correction (i.e., a reduction in false negatives when using PIMMS). For the fMRI runs with head movement, the number of activated voxels detected for data processed using Method A+MP as a percentage of those for Method B was 64%, 75% and 33% for Subjects 1 to 3 respectively which could therefore be interpreted as a reduction in false negatives of 36%, 25% and 67% respectively. The corresponding maps of T‐scores on the right of Figure 6 also demonstrate the reduction in significantly activated voxels for Method A+MP compared with the PIMMS correction (Method B). One would expect that for the run without head movement, including the motion parameters would lead to minimal false negative activations. However, in the presented data, including the motion parameters also reduced the number of significantly activated voxels for Subject 2. Notably for this subject, differences between tSNR_A_ and tSNR_B_ were also greater. Possible reasons for this are addressed in the discussion.

## DISCUSSION

A method has been presented (PIMMS) to model the phase changes in EPI time series using a GLM and to use the estimated maps of model parameters (i.e., rate of change of phase maps) to correct for motion‐related distortions and the corresponding variance in EPI magnitude time series. The method uses the phase information which is available with every EPI volume acquired.

### Validity of PIMMS Model

The PIMMS approach presented here assumes that phase changes in EPI time series occur as a result of shim heating and change in head position. Furthermore, it is assumed that the phase changes are relatively small, can be modeled as linear functions of time or head rotation and that the different effects are independent. The validity of the PIMMS model and hence these assumptions were assessed by examining how well the model explained the change of phase data and the spatial structure of the resulting model fit parameters and modeled variance.

In studies presented here, the PIMMS model was able to explain a significant amount of variance in measured phase changes (*p* < 0.001) in more than 84% of voxels in the brain for the experiment with excessive head movements and more than 94% of voxels for the more typical fMRI experiments. Visual inspection of the average change of phase and modeled effects, as a function of image volume number (i.e., Fig. 2c,f,i), illustrate the fit of the PIMMS model to the change of phase averaged over the whole brain.

In the first experiment, subjects made purposeful head movements of about ± 2–3° which are quite large for a typical fMRI study. These movements lead to mean distortion‐related voxel displacements of up to 0.5 voxels which supports the assumption that the phase changes are usually small (Fig. 3b,d,f), as shown in previous studies [Hutton et al., 2002]. Regions where the PIMMS model explained less than 50% of the total phase variance were located close to the axis of the head rotation (Fig. 3a,c,e). In these regions the impact of the head rotation on the field was much lower than at the brain periphery due to the higher field homogeneity. As a consequence, the estimated model fit parameters in these regions were closer to a value of zero and possibly of the same order as the noise in the phase change data. A similar effect was apparent for the estimation of the rate of change of phase with respect to rotation about the *y*‐axis because the estimated head rotations about the *y*‐axis were very small. The different spatial structure between the rate of change of phase maps suggested that the effects modeled by each term were relatively independent. However, some effects of the head rotations about the *x*‐axis are also visible in the other parameter maps. Furthermore, some spatial noise is apparent in the maps of rate of change of phase with respect to head rotation about the *y*‐axis and with respect to time. In particular some noise is apparent in regions of high vasculature. The negative impact of these effects on the PIMMS correction was minimized by using a spatially smoothed linear combination of the scaled parameter maps to perform the dynamic distortion correction. The source of the noise in the parameter maps and its effect on the PIMMS correction are addressed in the following section.

### Impact of PIMMS Correction

The data from Experiment 1 showed that motion‐related variance in the EPI magnitude time series was significantly reduced in peripheral regions and at some tissue boundaries after the PIMMS correction compared with standard realignment (Fig. 4). In corresponding regions the tSNR was also greater after the PIMMS correction. These results suggest that the PIMMS procedure was able to correct for the dynamic distortion effects which lead to stretching and compression of the brain in the EPI volumes.

The PIMMS correction will introduce some spatial smoothing as a result of the nonlinear resampling of each image into the space of the first image and this may reduce overall variance in the images compared to standard realignment. The impact of the correction was therefore assessed using preprocessed data after spatial smoothing using a Gaussian kernel with FWHM = 6 mm to equalize any smoothing effect between the PIMMS correction and standard realignment. Furthermore, since the comparisons between the tSNR maps (in Fig. 4c,f,i) show localized differences, it is unlikely that the reduction in variance in the PIMMS corrected data can be attributed to the minimal smoothing introduced by nonlinear resampling.

The PIMMS correction also increased motion‐related variance in a small number of small regions in the brain relative to the standard realignment (e.g., in Fig. 4b,e,h). There are a couple of possible reasons for this. First of all, it is possible that the PIMMS model fits the phase change data less well in these regions, or that overfitting occurs, leading to noise in the estimated parameter maps. This may be a result of the change of phase being very small and therefore of a similar scale to the noise in the phase data, perhaps due to susceptibility‐related signal loss or uncorrected phase discontinuities. Regularization of the model fitting could prevent this. Second, the relative displacement of tissue boundaries between the PIMMS corrected and the realigned data could result in a shift of small residual motion effects in the PIMMS corrected data to a region where the realigned data are artifact free. Overall the results in Figure 4 show that the PIMMS correction performed well in most of the brain without an obvious decrease in performance in regions where the PIMMS model described less than 50% of the variance of the phase change (i.e., compare with Fig. 3).

When PIMMS was applied to data from a typical fMRI study with and without small stimulus‐correlated head movements (i.e., Experiment 3), there were no significant differences between the magnitude of the parameters explaining the visual response for the PIMMS correction compared with standard realignment. This data demonstrate that the PIMMS approach could lead to a reduction in false negatives of more than 25% compared with the widely accepted approach which uses standard realignment and includes the motion parameters in the statistical model. For one of the three subjects, motion parameters also reduced the number of significantly activated voxels in the fMRI run without head movement. On inspection, the motion parameters estimated for this subject indicated that the subject moved less than 0.2° but the correlation coefficient between the motion and the visual task was 0.33. This may have been caused by signal changes from the visual activity introducing a bias into the image realignment algorithm as described by Freire and Mangin (2001). In general, the PIMMS approach may be a particularly valuable alternative method because the correction is based on motion‐related signal changes in the phase rather than the magnitude data. Although not studied here, one may assume that sensitivity to small BOLD activations would be improved in regions where the PIMMS correction reduced motion‐related variance and improved tSNR.

### Methodological Considerations

In general, retrospective dynamic distortion correction methods attempt to correct for a relatively small effect. Approaches such as those proposed in [Hahn et al., 2009; Hutton et al., 2002; Lamberton et al., 2007; Marques and Bowtell, 2005] which use a more direct measure of the phase acquired at each time point may introduce noise at the correction step. In contrast, modeling the phase changes in a GLM framework and hence parameterizing the effects, results in a correction at each time point that is constrained by the linear process and is therefore less likely to introduce noise. However, it should also be noted that the PIMMS method requires additional and accurate information in order to model the changes in the phase data, such as reliable motion estimates.

A measure of the static distortion present in the whole fMRI time series due to the B_0_ field inhomogeneities is not provided by the PIMMS model. However, the model for the change in distortion over time can be combined with other methods to correct for both the static and dynamic effects of distortion. For example, the parameter map resulting from fitting the constant term included in the PIMMS model could be combined with a phase image acquired at a different echo time to calculate a correction for static distortion effects which could be combined with the dynamic distortion effects.

The PIMMS approach as it is implemented here assumes that the initial estimation of motion parameters is negligibly affected by the changes in distortion from one time point to another. However, to account for possible inaccuracies in the initial estimation, the PIMMS corrected data were realigned and resliced in the final step of the PIMMS procedure. The standard deviation of the initial motion parameters averaged over all EPI time series acquired in Experiment 1, was reduced by 91.2% as a result of the PIMMS correction and 99% after the final realignment step following the PIMMS procedure (data not shown). In comparison, the standard deviation of the initial motion parameters was reduced by 98.6% as result of the standard realignment alone. The results suggest that the initial estimation of the motion parameters may be affected by differential distortions throughout the time series when there are large head movements. The final realignment step was therefore included in the PIMMS correction. In general, performing the whole PIMMS processing procedure (as outlined in Fig. 1) in an iterative fashion would relax this requirement.

In general it is difficult to demonstrate the impact of dynamic distortion correction in standard fMRI studies, since every effort is made to keep head movements to an absolute minimum. To demonstrate the effect of the PIMMS procedure, it was applied in an experiment where subjects were instructed to make relatively large head movements and acquisition parameters were untypical for an fMRI experiment. For example, in this experiment, the volume TR included a delay so that subjects could move their heads in the time between image acquisitions. In the absence of the delay between EPI volume acquisitions, large intra‐scan head movements will be less accurately estimated using rigid body realignment and since the PIMMS procedure considers each phase image volume as a single point in time, the goodness of fit of the PIMMS model may be reduced. Nevertheless, the linear modeling process employed by PIMMS is unlikely to introduce additional noise compared with standard realignment, as demonstrated by the data acquired in Experiment 3 where subjects made small stimulus‐correlated head movements.

Another motion‐related effect which gives rise to signal changes in EPI time series is due to the excitation history of the spin system (spin history effect [Friston et al., 1996]). This effect occurs because for a given image volume, the current magnetic state of the system depends on the previous magnetic states if the spin system has no time to return to equilibrium before the next excitation pulse occurs. Therefore a change of the object position in one image will have an impact on the intensity in subsequent image volumes. As a consequence of the long TR used in Experiment 1 with large head movements, spin history effects could be assumed to be minimal or nonexistent. However, in more typical fMRI studies, spin history effects will remain a source of variance [Muresan et al., 2005] even if motion and susceptibility‐related variance is reduced by the PIMMS method.

The size of the distortion effects caused by head motion studied here were quite small. In general these effects will be larger and more likely to introduce signal variance at higher field strengths [Hutton et al., 2011], unless accelerated imaging techniques are used which reduce the distortion effects. Accelerated imaging techniques such as SENSE‐EPI [Pruessmann et al., 1999], which allow for reduced EPI readout times, result in reduced susceptibility‐related image distortions compared with conventional EPI techniques. This has been demonstrated in fMRI studies using parallel imaging techniques [Preibisch et al., 2003; Schmidt et al., 2005].

In this study, raw data were acquired using a head transmit‐receive RF coil. It is possible that the change in phase of the RF field resulting from head motion may also affect the phase of the EPI signal. Although this dynamic RF phase change was not obvious in the data acquired here and to our knowledge has not been reported in the literature, it may become more significant at higher field strengths and could be investigated using B1 phase maps (e.g., [Metzger et al., 2008]). It is also important to note that for this study, phase and magnitude images were reconstructed from the raw data using a customized image reconstruction method. This method combines k‐space trajectory measurement, algebraic reconstruction and navigator echo correction to yield images with minimized Nyquist ghosts and without line artefacts [Josephs et al., 2000]. Using a customized image reconstruction has the advantage that all reconstruction steps are known and well controlled. In contrast, when phase images are reconstructed using manufacturer‐provided algorithms, hidden correction steps such as B_0_ drift correction, may affect the phase maps and therefore the estimation and interpretation of the PIMMS model. Furthermore, if PIMMS is applied to data acquired using multichannel receiver coils, particular attention to the combination of phase information from the different channels is required, especially at higher field strengths because each coil has its own intrinsic phase variation, e.g., see [Chen et al., 2010; Hammond et al., 2008; Lu et al., 2008; Robinson et al., 2011; Robinson and Jovicich, 2011; Roemer et al., 1990].

### Possible Extensions to the PIMMS Framework

A strength of the PIMMS framework is the flexibility that allows for other terms to be included in the model. The implementation presented here assumed that changes in phase were mainly caused by rotations about the x and y axes and the model therefore included these terms as well as the linear function of time to model the passive shim heating effect. Although head translations and rotations about the z axis should have a minor effect on the field, significant movement away from the initial position where the head was optimally shimmed could also lead to changes in the field. This was tested for the data presented here by including all six head motion parameters in the PIMMS model. The results showed that including the additional movement parameters did not improve the PIMMS model fit. In general, the six degrees of freedom for movement correction is a theoretical construct and moving the head independently along these degrees of freedom is very difficult. A singular value decomposition of movement parameters invariably yields one or two modes that account almost perfectly for all the movement. The remaining four movement parameters are therefore often highly correlated to the two first, and will contribute little or nothing to reducing the residual error and poorly condition the inversion step used to estimate the GLM. It should also be mentioned at this point that the relevant motions may be site and experiment specific.

Higher order frequency drifts could be modeled for the sensitive detection and characterization of phase deviations which could be useful in quality assurance procedures. This may be of particular importance when using other equipment during an fMRI study such as EEG [Hinterberger et al., 2004], TMS or electrical stimulus equipment (e.g., leakage currents occurring in TMS, [Weiskopf et al., 2009]). Furthermore, other explanatory variables that impact on phase data such as physiological effects [Hutton et al., 2011; Van De Moortele et al., 2002] can also be included in the PIMMS model [Hutton et al., 2008]. The framework could also be extended to allow for slice‐specific models and hence the inclusion of effects with a higher temporal resolution. For example, information from the monitoring of jaw or body movement, e.g., [Keliris et al., 2007], and also MR image independent high temporal resolution estimates of head motion from optical tracking systems [Zaitsev et al., 2006] could be included in the PIMMS model.

Another possible extension of the presented PIMMS approach is an implementation to perform distortion correction in real time. This would require that the GLM is solved for each volume as it is acquired rather than being calculated for a whole time series retrospectively. This would be possible by exploiting data processing and model fitting methods employed for calculating functional activation maps in real time, e.g., [Cox et al., 1995; Pollock, 1999; Weiskopf et al., 2003;Weiskopf et al., 2007].

## CONCLUSIONS

A new method (PIMMS) has been presented to linearly model phase changes in EPI data and to use the estimated parameters to perform a dynamic geometric distortion correction. The results of a proof of concept study demonstrate the validity of the PIMMS model and show that the PIMMS correction can reduce motion‐related variance and improve temporal SNR. The method works with any standard EPI sequences with access to the phase as well as the magnitude data.
